# 84357 A TL1 Team Approach to Integrating Mathematical and Biological Models to Target Myeloid-Derived Immune Cells in Glioblastoma

**DOI:** 10.1017/cts.2021.456

**Published:** 2021-03-30

**Authors:** Gregory P. Takacs, Hannah Anderson, Christian Kreiger, Defang Luo, Libin Rong, Tracy Stepien, Jeffrey K. Harrison

**Affiliations:** 1 Department of Pharmacology & Therapeutics, University of Florida College of Medicine; 2 Department of Mathematics, University of Florida College of Arts and Sciences

## Abstract

ABSTRACT IMPACT: Predicting therapeutic responses in GBM. OBJECTIVES/GOALS: The goal of this team approach is to integrate mathematical models of glioblastoma (GBM) infiltrating myeloid cells that contribute to the immunosuppressive phenotype in glioma with experimental data to predict therapeutic responses to combined chemokine receptor and immune checkpoint blockade. METHODS/STUDY POPULATION: Orthotopic murine KR158-luc gliomas were established in fluorescent reporter CCR2WT/RFP CX3CR1WT/GFP mice. Subsequently, an anti-CD31 injection was administered to label the vasculature. Fluorescent imaging and quantification of anti-CD3 stained sections were performed on a range of tumor sizes to acquire vasculature, tumor, T cell, and myeloid cell densities. In parallel, a system of ordinary differential equations was formulated based on biological assumptions to evaluate the change over time of tumor cells, T cells, and infiltrating myeloid cells. The model was then refined and validated by experimental results. RESULTS/ANTICIPATED RESULTS: Fluorescent imaging and quantification revealed a correlation between tumor size and abundance of (CX3CR1+, CCR2-) and (CX3CR1+, CCR2+) myeloid cell populations in the tumor microenvironment. The density of these cell populations and vasculature remained constant as the tumors increased in size. Computer simulations of the mathematical model will predict tumor, myeloid, and T cell dynamics. These simulations will be particularly useful to uncover information regarding myeloid cell dynamics, such as cell entry time into the tumor microenvironment. Parameter sensitivity analysis of the model will inform us of the biological processes driving these tumor-immune cell dynamics. DISCUSSION/SIGNIFICANCE OF FINDINGS: GBM is a challenge as current intervention are ineffective. This study improves the understanding of glioma infiltrating myeloid cells and their impact on tumor progression. The data will serve as a basis for quantitatively predicting therapeutic responses of a novel combination treatment.

